# Effects of organic acids on purple potato wine brewing: methanol, higher alcohols and flavor

**DOI:** 10.1016/j.fochx.2026.104211

**Published:** 2026-07-13

**Authors:** Xiaolong Yuan, Nan Chen, Zhengyun Wu, Siqi Liu, Xin Xiang, Wenxue Zhang, Gomi Katsuya

**Affiliations:** a College of Biomass Science and Engineering*,* Sichuan University*,* Chengdu 610065*,* China; b Laboratory of Fermentation Microbiology, Graduate School of Agricultural Science*,* Tohoku University*,* Sendai 980-8572*,* Japan

**Keywords:** Purple potato wine, Organic acids, Higher alcohols, Methanol, Transcriptomics

## Abstract

Methanol and higher alcohols pose health risk in many alcoholic beverages. In this study, the effects of adding organic acids on the fermentation and methanol and higher alcohol levels in purple potato wine were investigated. To explore the mechanisms underlying the effects of organic acids, a transcriptome analysis and enzyme activity assays were performed. The addition of acetic, lactic, and citric acids increased the types and contents of volatile substances such as esters and acids, and improved the taste and flavor of wine. All three organic acids effectively reduced methanol and higher alcohol contents in the wine, with acetic acid exhibiting the most pronounced effect. The optimal concentration of acetic acid was 0.2%, at which methanol and higher alcohol levels were reduced by 17.72% and 31.34%, respectively. Although a higher concentration of 0.25% resulted in a slightly greater reduction, it led to excessively high total acidity, which may adversely affect wine quality.Transcriptome analyses revealed that the addition of acetic and lactic acid increased transcript levels of genes encoding alcohol acetyltransferase and alcohol acyltransferase, which are involved in ester synthesis, by 77.88% to 96.03%. Conversely, the transcript levels of genes encoding pyruvate decarboxylase and aldehyde reductase, associated with alcohol synthesis, decreased by 26.88% to 65.74%. Furthermore, the activity of pectinesterase, linked to methanol formation, decreased by 12.47% to 23.08%. This study contributes to quality improvement of potato wine and other similar fermented alcoholic drinks

## 1 Introduction

As a fermented wine made from purple potatoes, purple potato wine (PPW) is highly favored by consumers for its beautiful color, unique flavor, and high contents of anthocyanins, vitamins, and other nutrients. Purple potatoes have high pectin and amino acid contents; these substances are easily converted to methanol and higher alcohols during the winemaking process and form the main health risk factors in wine (Saigusa et al., 2007).

Methanol, a common health risk factor in alcohol, may trigger various symptoms, such as headache and nausea, even when consumed in trace amounts, and can lead to blindness in severe cases (Bordet et al., 2021). Consequently, a number of countries have established limits. For example, standards stipulate that the methanol content of fruit-based distilled spirits should be less than 10.00 g/L in the European Union (European Parliament and of the Council (EU), 2019), less than 7.00 g/L in the United States and Australia (Bindler et al., 1988), and less than 400 mg/L in China (Botelho et al., 2020). In fruit wine fermentation systems, methanol formation is primarily attributed to the enzymatic hydrolysis of pectin catalyzed by pectin methylesterase (PME), which releases methanol from methyl-esterified pectin in plant cell walls. Therefore, PME-mediated pectin degradation is considered the major and most widely accepted pathway for methanol production in purple potato wine fermentation(Bordet et al., 2021).

Higher alcohols are alcohols containing more than three carbon atoms, including isobutanol, isoamyl alcohol, n-butanol, n-propanol, active pentanol, and beta-phenylethanol (Zheng et al., 2020). These alcohols are necessary for flavor development but they can also cause off-flavors and headaches when present beyond a safe level (Huang et al., 2023). Higher alcohols are mainly produced by the amino acid catabolic pathway (Ehrlich pathway) and gluconeogenic pathway (Harris pathway). In the Ehrlich pathway, precursor amino acids are converted into corresponding higher alcohols by the action of decarboxylase, transaminase, and dehydrogenase. The Harris pathway is the process by which glucose is converted into α-keto acids through glycolysis and the tricarboxylic acid cycle and then into corresponding higher alcohols through the action of ketoacid decarboxylase and dehydrogenase (El-Dalatony et al., 2019).

Common means of controlling the levels of methanol and higher alcohols in fermented wine include optimizing the brewing process (Ntuli et al., 2022), constructing yeasts (Li et al., 2020) that produce lower levels of methanol or higher alcohols through selection or genetic engineering (Hu et al., 2022; Liu et al., 2022), and adding exogenous substances (Liu, Gao, & Zhang, 2021). As the brewing process is usually constrained by many factors, its substantial adjustments are difficult; the application of genetically engineered bacteria in food production is strictly limited, and the selection and breeding of favorable yeast strains usually takes a long time. Under this circumstances, adding exogenous substances to reduce the methanol and higher alcohol contents is an convenient solution. Adding organic acids (gallic acid, coumaric acid, citric acid and acetic acid, for example) is reported to be able to reduce methanol and higher alcohol contents in wine (Gong, 2003; Hou et al., 2008). However, most of these studies primarily focused on phenotypic changes in volatile compounds, while the underlying molecular regulatory mechanisms remain largely unclear.

In this context, the present study focuses on purple potato wine fermentation and integrates transcriptomic analysis to systematically investigate the regulatory effects of organic acids on aroma-related metabolic pathways. This approach provides new insights into the molecular mechanisms underlying aroma modulation in a non-traditional fruit wine system.

## 2 Materials and methods

### 2.1 Main experimental materials and reagents

Purple sweet potatoes were purchased from Wal-Mart Supermarket in Wuhou, Chengdu, and *Saccharomyces cerevisiae* SY was purchased from Angel Yeast Co. Ltd. Glucoamylase (50,000 U/g) and α-amylase (10,000 U/g) were purchased from Henan Xingxing Food and Chemical Biotechnology Company.

### 2.2 Brewing of PPW

Fresh purple potatoes were cleaned and steamed at high temperatures for 20 min. They were subsequently cooled and turned into pulp with distilled water at a ratio of 1 g: 0.4 mL. Then, 0.15% α-amylase and 0.2% glucoamylase were added, and enzymatic digestion was allowed to take place at 65 °C for 2 h, before the mixture was placed in a press. The composition of the purple potato juice was adjusted by adding 13% sugar, 0.025% potassium metabisulfite, 0.03% sodium erythorbate, and 0.2% citric acid.

Acetic acid, lactic acid, and citric acid were added at mass concentrations of 0.1% - 0.25%, respectively. 0.1% of activated *Saccharomyces cerevisiae* were inoculated for fermentation at a constant temperature of 25 °C for 8 days. Samples were taken at 2-day intervals for the determination of physicochemical indices and the contents of higher alcohols and methanol. Based on the concentration of exogenous substances added, the groups supplemented with acetic acid were numbered sequentially as AA-1, AA-2, AA-3, and AA-4 (0.1%, 0.15%, 0.2%, and 0.25%, respectively), the groups supplemented with lactic acid were numbered LA-1, LA-2, LA-3, and LA-4, and the groups supplemented with citric acid were numbered CA-1, CA-2, CA-3, and CA-4. The control group without the addition of organic acids was marked as CK.

### 2.3 Measurement of physicochemical indices

National standard (*GB/T 15038–2006 analytical methods of wine and fruit wine. Beijing, China,* 2006) was referenced to determine the pH and total acidity during the fermentation process. The content of reducing sugars was determined using the 3,5-dinitrosalicylic acid (DNS) colorimetric method (Asquieri et al., 2019). The alcohol content was determined using an alcoholometer. The anthocyanin content was determined using a colorimetric method. Lastly, pectinesterase enzyme activity was determined using the pH-stat method (Moens et al., 2021).

### 2.4 Determination of volatile components

The content of volatile flavor compounds was determined using gaschromatography-mass spectrometry (TSQ 9610, Thermo Fisher Scientific Inc., USA). Five milliliters of PPW and 10 μL of 100 μg/L 2-octanol (internal standard) were added to an extraction bottle. The mixture was equilibrated in a 60 °C water bath for 10 min. The extraction head was then inserted, and extraction took place for 45 min, followed by 5 min of desorption at the injection port. The chromatographic column was VF-WAXms (30 m × 0.25 mm × 0.25 μm) with a split ratio of 1:20. The temperature program was as follows: the mixture was held at 40 °C for 5 min, increased to 100 °C at a rate of 4 °C/min, and increased to 230 °C at a rate of 6 °C/min. Afterwards, it was held at 230 °C for 2 min. The injector temperature was 250 °C, and the detector temperature was 270 °C. For electron ionization (EI), the electron energy was set at 70 eV, with an ion source temperature of 250 °C. A solvent delay of 2 min was implemented, and the mass spectrometer scanned over a range of 50 to 450 amu (*m*/*z*). The content of volatile compounds was calculated based on the concentration of the internal standard, 2-octanol (Liu, Ma, et al., 2021).

### 2.5 Determination of methanol and higher alcohol contents

For the determination of methanol and higher alcohol contents, the method of Zheng et al. (2020) was used with slight modifications. After distillation of the PPW, gas Chromatograph (Trace 1300, Thermo Fisher Scientific Inc., USA) was used for analyses. The chromatographic column employed was a DB-WAX (30 m × 0.32 mm × 0.25 μm). The GC temperature program began at an initial temperature of 40 °C, held for 1 min, then increased to 70 °C at a rate of 4 °C/min and held for 1 min, followed by an increase to 220 °C at 10 °C/min, and finally held for 1 min. The split ratio was 1:20, with an injector temperature of 200 °C and a detector temperature of 230 °C. Nitrogen was used as the carrier gas with a flow rate of 35 mL/min. The hydrogen flow rate was 40 mL/min, and the air flow rate was 350 mL/min. The analytical method was validated in terms of linearity, limits of detection (LOD), limits of quantification (LOQ), recovery, and repeatability. The detailed validation results are presented in Supplementary Table S2.

### 2.6 Transcriptome analysis and qRT-PCR validation

RNA was extracted from CK, AA-3, and LA-3 samples on day 4 of fermentation, representing a metabolically active stage associated with aroma formation. RNA integrity was assessed by 1% agarose gel electrophoresis. rRNA was removed using a magnetic bead-based kit, and mRNA was purified for library construction.

RNA was fragmented and used for first- and second-strand cDNA synthesis to generate double-stranded cDNA. After end repair, A-tailing, and adapter ligation, the libraries were PCR-amplified and sequenced on an Illumina platform (Majorbio Bio-pharm Technology Co., Ltd., Shanghai, China). Raw reads were filtered to obtain clean reads, which were aligned to the *Saccharomyces cerevisiae* reference genome using HISAT2. Gene expression levels were calculated as FPKM, and differentially expressed genes (DEGs) were identified using DESeq2 with thresholds of |log2(fold change)| ≥ 1 and adjusted *p*-value (FDR) < 0.05. Each experimental group contained three biological replicates.

To validate RNA-seq results, qRT-PCR was performed on selected DEGs (*ACS1*, *ATF1*, *EHT1*, *GCV1*, *PDC1*, *PDC6*, *ADH2*, and *ILV2*). Total RNA was reverse-transcribed into cDNA using a commercial kit according to the manufacturer's instructions. qRT-PCR was conducted on an ABI 7500 Real-Time PCR system (Applied Biosystems, USA) using SYBR Green chemistry. The thermal cycling conditions were as follows: 95 °C for 30 s, followed by 40 cycles of 95 °C for 5 s and 60 °C for 34 s. Relative gene expression was calculated using the 2^−^ΔΔCt method, with *ACT1* as the internal reference gene and CK as the calibrator. All reactions were performed in triplicate.

### 2.7 Sensory evaluation

Sensory evaluation was performed by a trained panel consisting of six experienced assessors. Prior to evaluation, all panelists received training on aroma attribute recognition and scoring procedures to ensure consistency. The evaluation was conducted in a standardized sensory laboratory under controlled conditions (temperature 22 ± 1 °C, relative humidity 50–60%, and low-light environment). Samples were presented in a randomized order using a blind-coded system to eliminate bias. The scoring criteria refer to the national standard (*GB/T 15038–2006 analytical methods of wine and fruit wine. Beijing, China,* 2006), and the total score is calculated as 100 points, with the highest score and the lowest score removed from each group, and the average score is the final score. Ethical statement: Ethical approval was not required for this sensory evaluation because the study involved voluntary sensory assessment of food products and posed no more than minimal risk to participants. All procedures complied with institutional guidelines for protecting participants' rights and privacy. Written informed consent was obtained from all participants prior to the sensory evaluation.

It should be noted that sensory evaluation in this study was conducted with a relatively small panel size (*n* = 6), which may limit the statistical robustness of sensory conclusions. Future studies with larger and more diverse panels are needed to further validate the sensory results.

### 2.8 Statistical analysis

All experiments were performed in triplicate, and results were expressed as mean ± standard deviation (SD). Statistical analysis was conducted using OriginPro 2023 (OriginLab Corporation, Northampton, MA, USA). One-way analysis of variance (ANOVA) was used to evaluate differences among multiple groups. When significant differences were detected, Tukey's multiple range test was applied for post hoc comparisons. A value of *p* < 0.05 was considered statistically significant. Different lowercase letters indicate significant differences (p < 0.05).

## 3 Results and discussion

### 3.1 Effects of organic acid addition on PPW fermentation

**Fig. 1:**
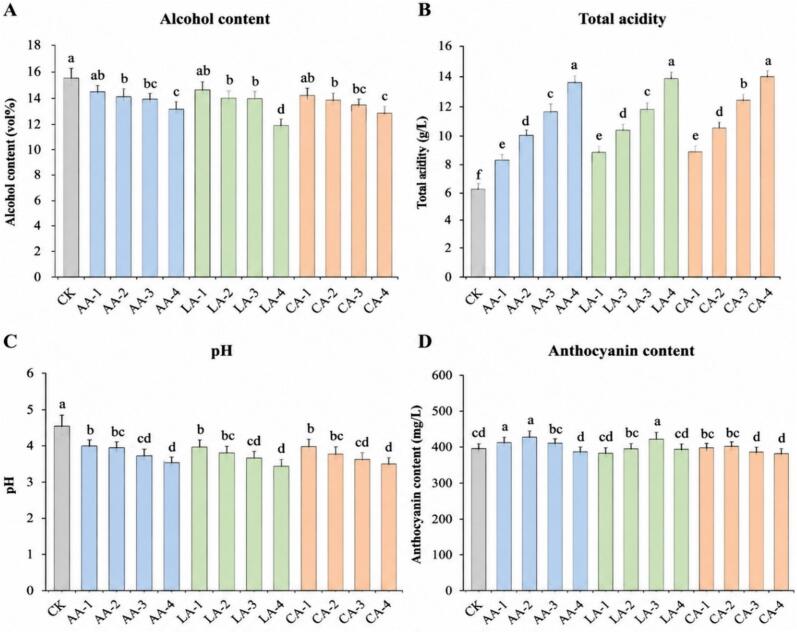
Alcohol content, total acidity, pH value, and anthocyanin content of PPW after fermentation with three organic acid additions.

The fermentation processes for all groups with the addition of three organic acids were generally similar to that of the control group (Supplementary Fig. S1). As shown in Fig. 1, the total acid content increased with the rising concentration of added organic acids, while the anthocyanin content also showed a slight increase. Except for AA-4, LA-4, CA-3, and CA-4, the total acid contents of the remaining experimental groups met the requirements of the Chinese national standard (*NY/T 1508–2007 green food fruit wine. Guangzhou, China,* 2007). After fermentation with added organic acids, the pH of the PPW decreased, and the alcohol content was slightly lower than that of the control group. The AA-4 and LA-4 groups showed greater decreases in alcohol contents than those in other groups (Supplementary Fig. S1). This showed that high concentrations of organic acids inhibited the growth and metabolism of yeast.

Excluding the samples with higher total acid contents (AA-4, LA-4, CA-3, and CA-4), 50 volatile components were detected in the remaining seven wine sample types (Appendix S1), including alcohols (8), esters (20), terpenes (3), aldehydes (3), ethers (2), phenols (3), ketones (4), acids (4), and others (3). There were more types and higher contents of volatile components, such as esters, terpenes, and acids, in PPW with the addition of organic acids than in the control group. The contents of some esters increased significantly. For example, the content of ethyl acetate increased from 2401.6 μg/L to 44,774.91 μg/L in the AA-3 group. In the CA-2 group, the content of isoamyl acetate increased from 1532.5 μg/L to 9242.8 μg/L. This can partly be explained by the fact that organic acids, such as acetic acid, are precursors of ester components.

**Fig. 2:**
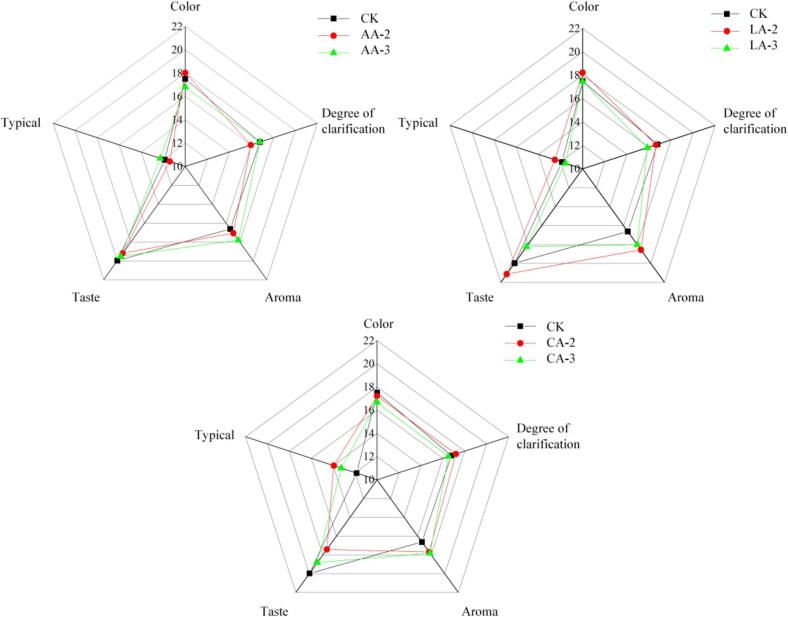
Sensory evaluation diagram of PPW fermented with the addition of three organic acids.

The sensory evaluation results for PPW fermented with added organic acids are shown in Fig. 2. The LA-2 wine sample had a rich aroma and a mellow, smooth taste, scoring the highest in the overall sensory evaluation among all samples. This can be attributed to the lower higher alcohol content and a noticeable reduction in bitter taste in LA-2. The second highest score was obtained for AA-3, which showed notable advantages with respect to aroma and taste, with significantly higher levels of esters, such as ethyl acetate and isoamyl acetate, compared with those of other wine samples.

### 3.2 Effects of organic acid on methanol and higher alcohol production during the fermentation of PPW

**Fig. 3:**
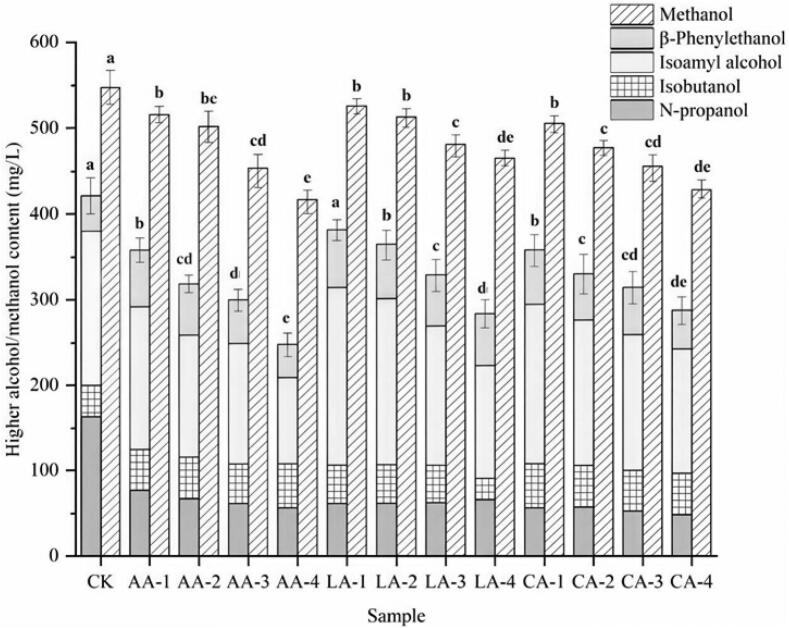
Methanol and higher alcohol contents in PPW fermented with the addition of three organic acids.

The methanol content increased gradually during the fermentation and stabilized after the fourth day (Supplementary Fig. S2). The final methanol level in the control group was 547.2 mg/L, exceeding the 400 mg/L limit set by Chinese national standard (*GB/T 15037–2006 Wine. Beijing, China,* 2006). The addition of all three organic acids reduced methanol formation, with more pronounced effects as the concentration increased (Fig. 3). In particular, the methanol content in the AA-3 group sample decreased by 17.72%.

The higher alcohol content in PPW fermentation rose rapidly from 0 to 2 days and stabilized after the fourth day (Supplementary Fig. S2). This is similar to the report of Yu et al. (2023). After fermentation, the higher alcohol content in the control sample was 404.95 mg/L. The reductions in higher alcohol contents increased with increasing concentrations of the three organic acids. Acetic acid had the best effect on reducing total higher alcohols in PPW, with a range of 11.47% to 38.52% (Fig. 3). After adding organic acids, the n-propanol content in PPW decreased by up to 70.1% and the isoamyl alcohol content decreased by up to 44.1%; significant reductions in isobutanol and β-phenethyl alcohol, however, were not detected. Previous studies have shown that the higher alcohol content in apple wine fermented with the addition of citric acid can be reduced by 9.6% to 28.1% (Gong, 2003). The reductions in higher alcohol contents in our study were much greater. A possible reason for this is that we used higher concentrations of organic acids.

### 3.3 Effects of adding acetic and lactic acids on gene transcription during fermentation of PPW

To investigate the underlying mechanisms of organic acid supplementation during the fermentation process, transcriptome analysis was performed on the AA-3 and LA-3 treatment groups, which achieved a better balance between sensory quality and methanol reduction, as well as the control group (CK), followed by validation using qRT-PCR (See Fig. 5, Fig. 6). A Venn diagram and volcano plot of differentially expressed genes (Supplementary Fig. S3) showed that AA-3/CK had 5203 common genes and 2161 differentially expressed genes. Among these, 1330 genes were upregulated, while 831 genes were downregulated in AA-3 compared with levels in CK. LA-3/CK had 5345 common genes and 1058 differentially expressed genes. Among these, 759 genes were upregulated, while 299 genes were downregulated. The addition of acetic acid had a greater impact on gene transcription levels of yeast than that of lactic acid. KEGG functional annotation (Supplementary Fig. S4) showed that the differentially expressed genes in samples fermented with added acetic acid and lactic acid were mainly involved in metabolism and genetic information processing. With respect to metabolic functions, the differentially expressed genes in AA-3 and LA-3 samples were mainly involved in amino acid metabolism and carbohydrate metabolism.

**Fig. 4:**
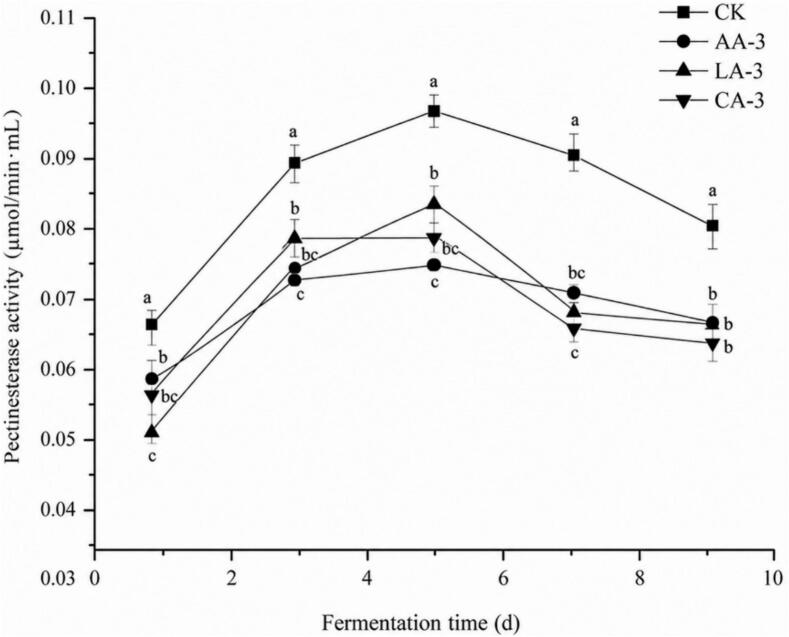
Pectin esterase activity during the fermentation with the addition of three organic acids in PPW.

**Fig. 5:**
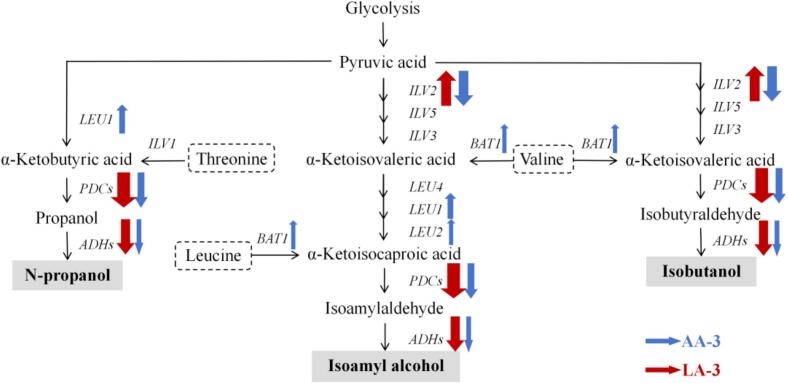
Transcript levels of key genes in the n-propanol, isobutanol, and isoamyl alcohol production pathways in fermentation with acetic acid and lactic acid. The width of the arrows are proportional to transcription levels.

**Fig. 6:**
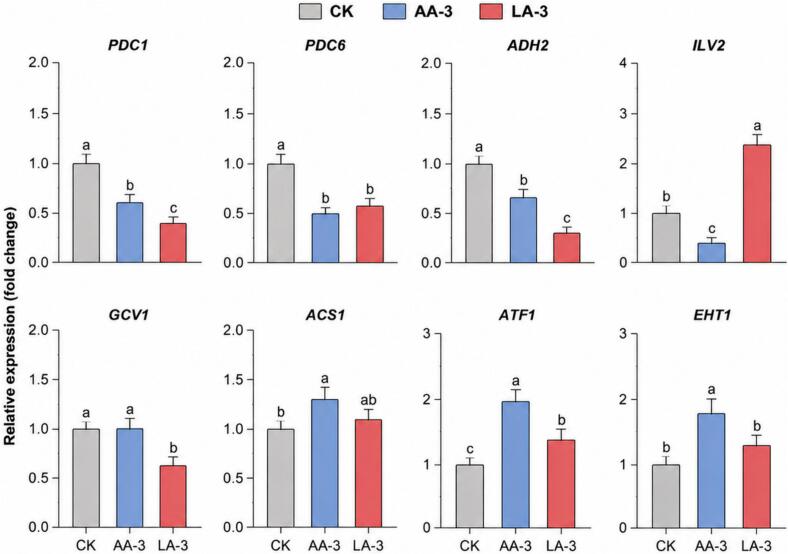
qRT-PCR validation of key genes involved in higher alcohol and aroma metabolism.

Ester compounds in wine are mainly produced by the catalysis of alcohol acetyltransferase/alcohol acyltransferase, with acetyl-CoA/acyl-CoA and ethanol as substrates (Scott et al., 2023). In the AA-3 group, the transcript level of the *ACS1* gene, which encodes acetyl-CoA synthetase, was 37.01% higher, the transcript level of the *ATF1* gene, which encodes alcohol acetyltransferase, was 96.03% higher, and the transcript level of the *EHT1* gene, which encodes alcohol acyltransferase, was 77.88% higher than corresponding levels in the control group. These results are consistent with the significantly higher levels of ester compounds in the AA-3 group than in other wine samples.

For LA-3 sample, the *GCV1* gene, which encodes the aminomethyltransferase that catalyzes glycine metabolism to produce methanol, was downregulated by 37.58%. A previous study (Xiong, 2015) has shown that fermentation using brewing yeast with *GCV1* gene knockout can reduce the methanol content in wine by 11.1%. In this study, the reduction in the methanol content in the LA-3 group may be related to the downregulation of *GCV1*. However, *GCV1* transcript levels were not reduced in the AA-3 group during fermentation.

In the transcriptome analysis, genes encoding the pectinesterase were not annotated. Based on searches against the UniProt database, pectinesterase is mainly distributed in plants, fungi (*Aspergillus*), and bacteria, and no pectinesterase sequences have been found in yeast. However, experimental studies have shown that some yeast strains possess pectinesterase activity (Haile & Kang, 2019). Although the yeast strain used in this study does not possess intrinsic PME activity, measurable PME activity was detected in the fermentation system. This phenomenon can be attributed to the endogenous PME present in purple potato raw materials, which is released during tissue disruption and fermentation processes. In our study, pectinesterase activity during the brewing showed an initial increase, followed by a decrease and was highest on the fourth day of fermentation (Fig. 4), which corresponds roughly to the changes in the methanol content during PPW fermentation (Supplementary Fig. S2). The addition of all three organic acids reduced pectinesterase activity significantly. In particular, pectinesterase activity was lower in the AA-3 group during the fermentation process, corresponding to the large reduction in the methanol content. Adding acidic substances was reported to reduce pectinesterase activity (Miljić et al., 2016). Pectinesterase is an extracellular enzyme with an optimum pH of approximately 4.5 (Haile & Ayele, 2022). The pH during our fermentation process with added organic acids was 3.36–4.16, which can directly inhibit pectinesterase activity.

Regarding the key enzymes for higher alcohol production, pyruvate decarboxylase (PDCs) and alcohol dehydrogenases (ADHs), the transcript level of the *PDC1* gene of LA-3 was 60.65% lower than that in the control group. For AA-3 and LA-3, transcript levels of the *PDC6* gene were decreased by 43.69% and 41.03% respectively, and *ADH2* transcript levels were decreased by 26.88% and 65.74% respectively. Notably, the transcript level of the *ILV2* gene encoding acetolactate synthase decreased by 59.71% for AA-3, but increased by 141.95% for LA-3. Previous study showed. That knocking out the *ILV2* gene in yeast reduced the isoamyl alcohol content in wine by greater than 20% (Li et al., 2020). The different directions of regulation in *ILV2* gene transcript levels may explain, in part, why the higher alcohol content in the AA-3 sample was lower than that in the LA-3 sample.

Overall, The downregulation of *PDC1*/*PDC6* and *ADH2* suggests a reduced carbon flux toward fusel alcohol formation. *ILV2* plays a key role in the biosynthesis of branched-chain amino acids, and the differential expression of this gene between AA-3 and LA-3 further explains the differences in higher alcohol accumulation. In addition, *ACS1*, *ATF1*, and *EHT1* are involved in regulating acetyl-CoA supply and ester biosynthesis, thereby competing for carbon resources with higher alcohol formation pathways. This metabolic competition between ester formation and higher alcohol production provides a reasonable explanation for the observed differences in aroma profiles.

## 4 Conclusion

The addition of organic acids increased the types and contents of volatile substances, such as esters, terpenes, and acids. PPW fermented with 0.2% acetic acid had good sensory characteristics, low higher alcohol and methanol contents. Adding acetic acid resulted in significant increase of the transcript levels of key enzyme genes in the ester synthesis pathway, such as alcohol acetyltransferase and alcohol acyltransferase, increased significantly. Adding acetic acid and lactic acid led to significant decrease of the transcript levels of key enzyme genes in the higher alcohol synthesis pathway. The addition of all the three organic acids inhibited the activity of pectinesterase, in the methanol synthesis pathway (pectin degradation pathway). Among all treatments, the LA-2 group achieved the highest sensory score. Notably, the extreme acidity in the CA-3 and CA-4 treatment groups precluded reliable volatile compound analysis. Additionally, transcriptomic analysis was limited to the AA-3 and LA-3 groups. Therefore, the mechanistic elucidation of citric acid in this study remains limited. Overall, adding organic acids could increase the contents of ester substances in PPW and inhibit the production of higher alcohols and methanol by promoting the transcription of key enzymes in the ester synthesis pathway and inhibiting the transcription and activity of key enzymes in the higher alcohol and methanol pathways.

## CRediT authorship contribution statement

**Xiaolong Yuan:** Writing – review & editing, Writing – original draft, Methodology, Conceptualization. **Nan Chen:** Writing – original draft, Methodology, Conceptualization. **Zhengyun Wu:** Writing – review & editing, Resources, Project administration, Funding acquisition. **Siqi Liu:** Writing – review & editing, Methodology, Investigation, Data curation, Conceptualization. **Xin Xiang:** Supervision, Investigation, Data curation, Conceptualization. **Wenxue Zhang:** Supervision, Resources, Project administration. **Gomi Katsuya:** Supervision, Resources, Project administration.

## Declaration of competing interest

The authors declare that they have no known competing financial interests or personal relationships that could have appeared to influence the work reported in this paper.

## Data Availability

Data will be made available on request.
