# Supplementary material for: Effects of organic acids on purple potato wine brewing: methanol, higher alcohols and flavor
Source: Food Chem X. 2026 Jul 13;38:104211. doi: 10.1016/j.fochx.2026.104211 (PMC13400438; doi:10.1016/j.fochx.2026.104211)

**Appendix**


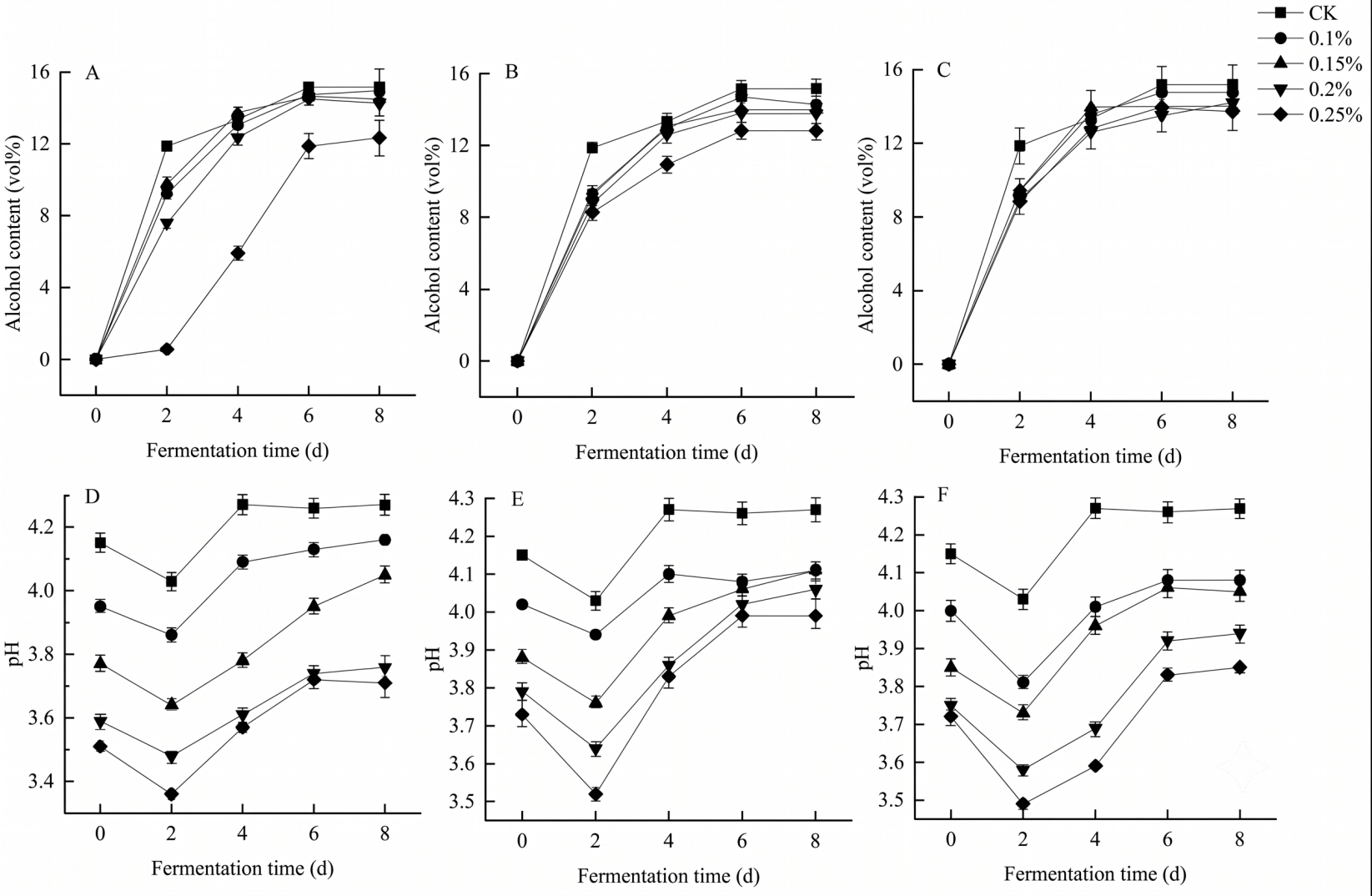


**Figure S1 Changes in the alcohol content (A: acetic acid, B: lactic acid, C: citric acid) and pH value (D: acetic acid, E: lactic acid, F: citric acid) during the fermentation of PPW with organic acids**

**Table S1 Contents (μg/L) of volatile components in PPW fermented with organic acids**

| Number | Volatile components | CK | AA-2 | AA-3 | LA-2 | LA-3 | CA-2 | CA-3 | Match |
| --- | --- | --- | --- | --- | --- | --- | --- | --- | --- |
|  | Alcohols |  |  |  |  |  |  |  |  |
| 1 | Isobutanol | 0.00 | 5333.59 | 7765.51 | 5347.98 | 2322.55 | 3758.30 | 0.00 | 92 |
| 2 | 1-Pentanol | 0.00 | 0.00 | 146.49 | 84.93 | 31.60 | 8061.32 | 0.00 | 84 |
| 3 | Phenylethanol | 319.98 | 93.85 | 180.00 | 133.85 | 91.70 | 122.31 | 105.02 | 95 |
| 4 | N-propanol | 14814.98 | 0.00 | 7422.24 | 4212.50 | 3500.71 | 15062.79 | 0.00 | 93 |
| 5 | Isoamyl alcohol | 262.46 | 104.37 | 0.00 | 0.00 | 0.00 | 84.04 | 159.72 | 90 |
| 6 | 2,3-Butanediol | 0.00 | 2362.65 | 11310.17 | 10230.11 | 7220.11 | 1795.15 | 0.00 | 88 |
| 7 | 1,2,3-Butanetriol | 0.00 | 53298.69 | 0.00 | 0.00 | 34823.82 | 0.00 | 0.00 | 81 |
| 8 | Panaxydol | 0.00 | 0.00 | 0.00 | 0.00 | 0.00 | 0.00 | 22075.96 | 77 |
|  | Esters |  |  |  |  |  |  |  |  |
| 9 | Ethyl acetate | 2401.45 | 1659.77 | 44774.91 | 1229.12 | 1021.74 | 1733.88 | 0.00 | 94 |
| 10 | Ethyl butyrate | 0.00 | 0.00 | 0.00 | 25646.47 | 24031.82 | 0.00 | 0.00 | 93 |
| 11 | Isoamyl acetate | 1532.53 | 7804.34 | 6150.59 | 4197.48 | 2109.22 | 9242.84 | 2902.71 | 92 |
| 12 | Ethyl caproate | 2443.23 | 13845.44 | 7530.93 | 4728.67 | 0.00 | 15319.48 | 6915.21 | 95 |
| 13 | 1-Methylheptyl acetate | 6438.50 | 24867.03 | 25111.08 | 22235.78 | 27557.67 | 30187.04 | 14698.07 | 83 |
| 14 | Ethyl caprylate | 221.79 | 1056.60 | 811.80 | 0.00 | 493.81 | 1966.13 | 683.17 | 91 |
| 15 | Ethyl caprate | 327.71 | 491.22 | 612.74 | 454.50 | 435.78 | 1288.66 | 622.17 | 90 |
| 16 | Phenylethyl acetate | 609.58 | 811.98 | 1113.42 | 572.23 | 576.97 | 1273.01 | 594.07 | 93 |
| 17 | Ethyl laurate | 3175.69 | 2759.07 | 3818.94 | 3014.31 | 2782.82 | 7883.51 | 3928.04 | 85 |
| 18 | Methyl palmitate | 0.00 | 0.00 | 0.00 | 0.00 | 0.00 | 0.00 | 1713.60 | 79 |
| 19 | Ethyl palmitate | 3418.92 | 3269.26 | 4539.15 | 3872.99 | 2214.53 | 6509.79 | 249.08 | 94 |
| 20 | 2-Hexyldecanoic acid methyl ester | 0.00 | 31009.65 | 75784.80 | 15738.96 | 0.00 | 0.00 | 0.00 | 82 |
| 21 | 10-Octadecenoic acid, methyl ester | 0.00 | 0.00 | 0.00 | 0.00 | 12286.29 | 0.00 | 0.00 | 81 |
| 22 | 13,16-Octadecadiynoic acid methyl ester | 35642.54 | 0.00 | 0.00 | 0.00 | 0.00 | 0.00 | 0.00 | 80 |
| 23 | Trans-9-Octadecenoic methyl ester | 4794.65 | 0.00 | 0.00 | 0.00 | 0.00 | 0.00 | 1021.43 | 81 |
| 24 | Methyl formate | 0.00 | 1390.72 | 0.00 | 0.00 | 0.00 | 2371.78 | 0.00 | 87 |
| 25 | Methyl 9-octadecenoate | 0.00 | 46127.98 | 0.00 | 0.00 | 0.00 | 0.00 | 0.00 | 82 |
| 26 | Ethyl 2-methyloctanoate | 0.00 | 0.00 | 0.00 | 0.00 | 28064.44 | 0.00 | 0.00 | 83 |
| 27 | Elaidic acid ethyl ester | 0.00 | 0.00 | 0.00 | 0.00 | 7377.65 | 0.00 | 1540.36 | 81 |
| 28 | Ethyl phenylacetate | 0.00 | 0.00 | 0.00 | 0.00 | 0.00 | 70885.86 | 0.00 | 96 |
|  | Aldehydes |  |  |  |  |  |  |  |  |
| 29 | 4-Phenylbutanal | 0.00 | 56051.16 | 187440.35 | 0.00 | 0.00 | 0.00 | 0.00 | 88 |
| 30 | 2-Bromooctadecanal | 0.00 | 0.00 | 0.00 | 0.00 | 0.00 | 872.34 | 0.00 | 76 |
| 31 | Acetal | 5563.13 | 1659.77 | 1605.94 | 1007.21 | 0.00 | 1282.02 | 0.00 | 84 |
|  | Ethers |  |  |  |  |  |  |  |  |
| 32 | Geranyl ethyl ether | 0.00 | 75856.16 | 0.00 | 0.00 | 0.00 | 0.00 | 0.00 | 86 |
| 33 | 4-Thiouridine disulfide | 0.00 | 30357.31 | 0.00 | 0.00 | 0.00 | 0.00 | 0.00 | 78 |
|  | Terpenes |  |  |  |  |  |  |  |  |
| 34 | Terpinen-4-ol | 0.00 | 0.00 | 0.00 | 0.00 | 25790.14 | 0.00 | 0.00 | 94 |
| 35 | Linalool | 0.00 | 0.00 | 0.00 | 12546.05 | 13288.34 | 427423.40 | 7315.80 | 96 |
| 36 | Alpha-terpineol | 0.00 | 0.00 | 0.00 | 8729.35 | 0.00 | 0.00 | 0.00 | 89 |
|  | Phenol |  |  |  |  |  |  |  |  |
| 37 | 2,4-Ditertbutyl phenol | 9141.50 | 3339.33 | 9053.83 | 6149.91 | 0.00 | 1538.51 | 0.00 | 83 |
| 38 | 2-Methoxy-phenol | 0.00 | 10566.18 | 10762.48 | 9964.69 | 0.00 | 0.00 | 0.00 | 85 |
| 39 | Thymol | 0.00 | 14589.29 | 0.00 | 8324.04 | 0.00 | 44607.74 | 0.00 | 94 |
|  | Ketone |  |  |  |  |  |  |  |  |
| 40 | 2-Octanone | 12906.01 | 23379.24 | 0.00 | 8712.67 | 0.00 | 0.00 | 0.00 | 93 |
| 41 | Damascenone | 0.00 | 0.00 | 35637.30 | 0.00 | 0.00 | 0.00 | 0.00 | 96 |
| 42 | Methyl vinyl ketone | 0.00 | 0.00 | 0.00 | 0.00 | 24374.78 | 0.00 | 0.00 | 82 |
| 43 | Pregnenolone | 0.00 | 0.00 | 0.00 | 0.00 | 4155.66 | 0.00 | 0.00 | 79 |
|  | Acids |  |  |  |  |  |  |  |  |
| 44 | Octanoic acid | 7912.91 | 7638.28 | 13553.60 | 0.00 | 0.00 | 0.00 | 0.00 | 94 |
| 45 | Lauric acid 2,3-diacetoxypropyl ester | 0.00 | 35785.80 | 0.00 | 0.00 | 0.00 | 0.00 | 0.00 | 83 |
| 46 | Phenylphosphinic acid | 0.00 | 0.00 | 12417.46 | 0.00 | 0.00 | 0.00 | 0.00 | 78 |
| 47 | 2,3-Dihydroxy-propanoic acid | 0.00 | 0.00 | 0.00 | 7842.04 | 0.00 | 0.00 | 0.00 | 82 |
|  | Others |  |  |  |  |  |  |  |  |
| 48 | 1,2-Dimyristoyl-sn-glycero-3-phosphocholine | 0.00 | 0.00 | 0.00 | 0.00 | 6845.61 | 0.00 | 0.00 | 77 |
| 49 | 7-methyl-benz-ofuran | 0.00 | 2725.23 | 3173.12 | 4107.81 | 0.00 | 0.00 | 0.00 | 83 |
| 50 | Styrene | 0.00 | 0.00 | 0.00 | 0.00 | 9687.11 | 0.00 | 0.00 | 95 |

**Table S2 Method validation parameters for volatile alcohols (GC–MS)**

| **Compound** | **Regression equation (y = ax + b)** | **R^2^** | **LOD (mg/L)** | **LOQ (mg/L)** | **Recovery (%)** | **RSD (%)** |
| --- | --- | --- | --- | --- | --- | --- |
| Methanol | y = 219.95x − 3.6126 | 0.9997 | 0.83 | 2.74 | 99.85 | 1.83 |
| N-Propanol | y = 33.032x − 6.3566 | 0.9995 | 1.21 | 3.99 | 98.31 | 2.16 |
| Isobutanol | y = 27.118x − 2.2177 | 0.9997 | 1.57 | 5.18 | 98.02 | 2.31 |
| Isoamyl alcohol | y = 27.278x − 2.0520 | 0.9997 | 1.55 | 5.12 | 101.87 | 2.25 |
| β-Phenylethanol | y = 29.196x − 1.4762 | 0.9998 | 1.03 | 3.40 | 99.73 | 1.08 |

**
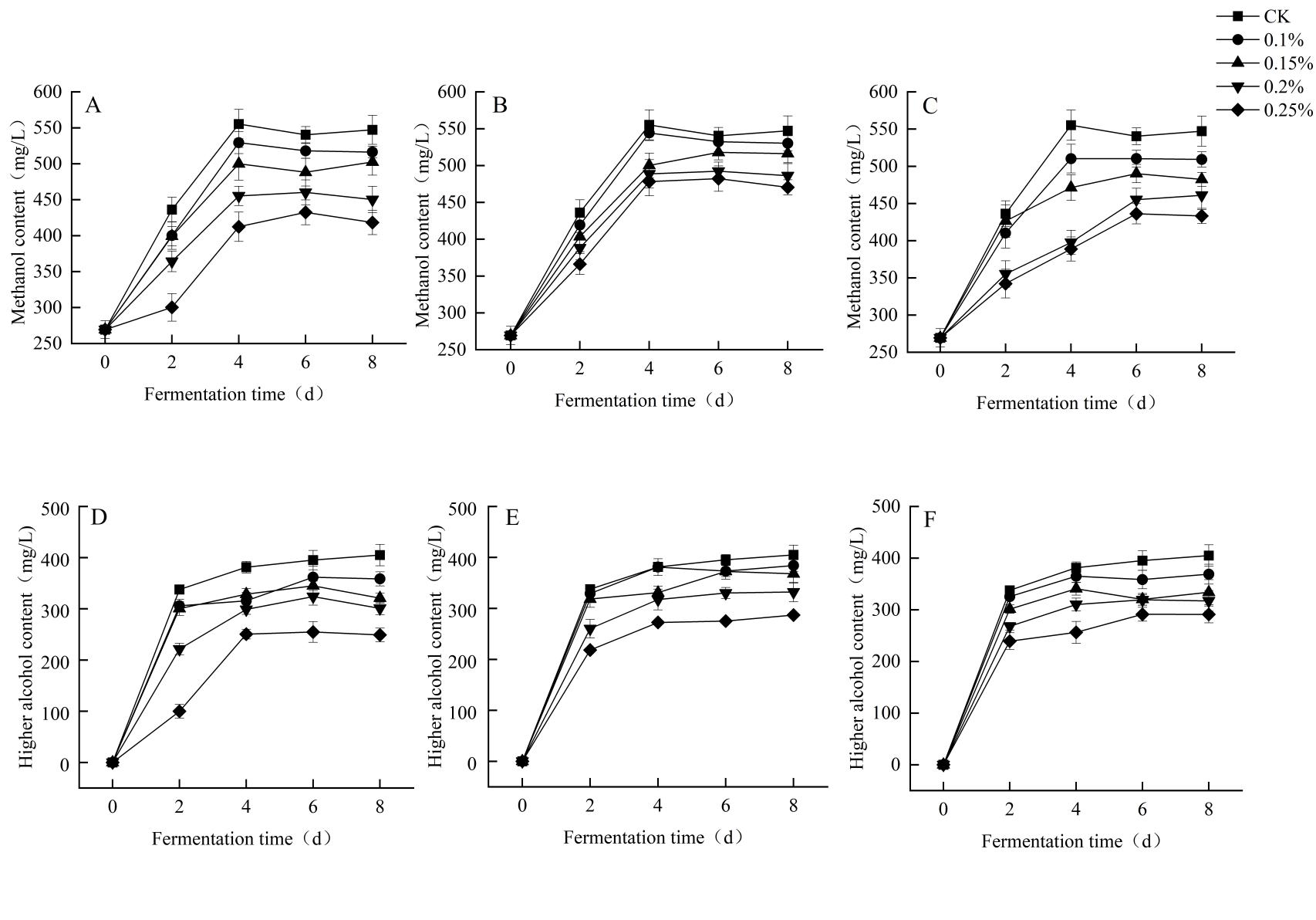
**

**Figure S2 Changes in the contents of methanol (A: acetic acid, B: lactic acid, C: citric acid) and higher alcohols (D: acetic acid, E: lactic acid, F: citric acid) during the fermentation of PPW with organic acids**

**
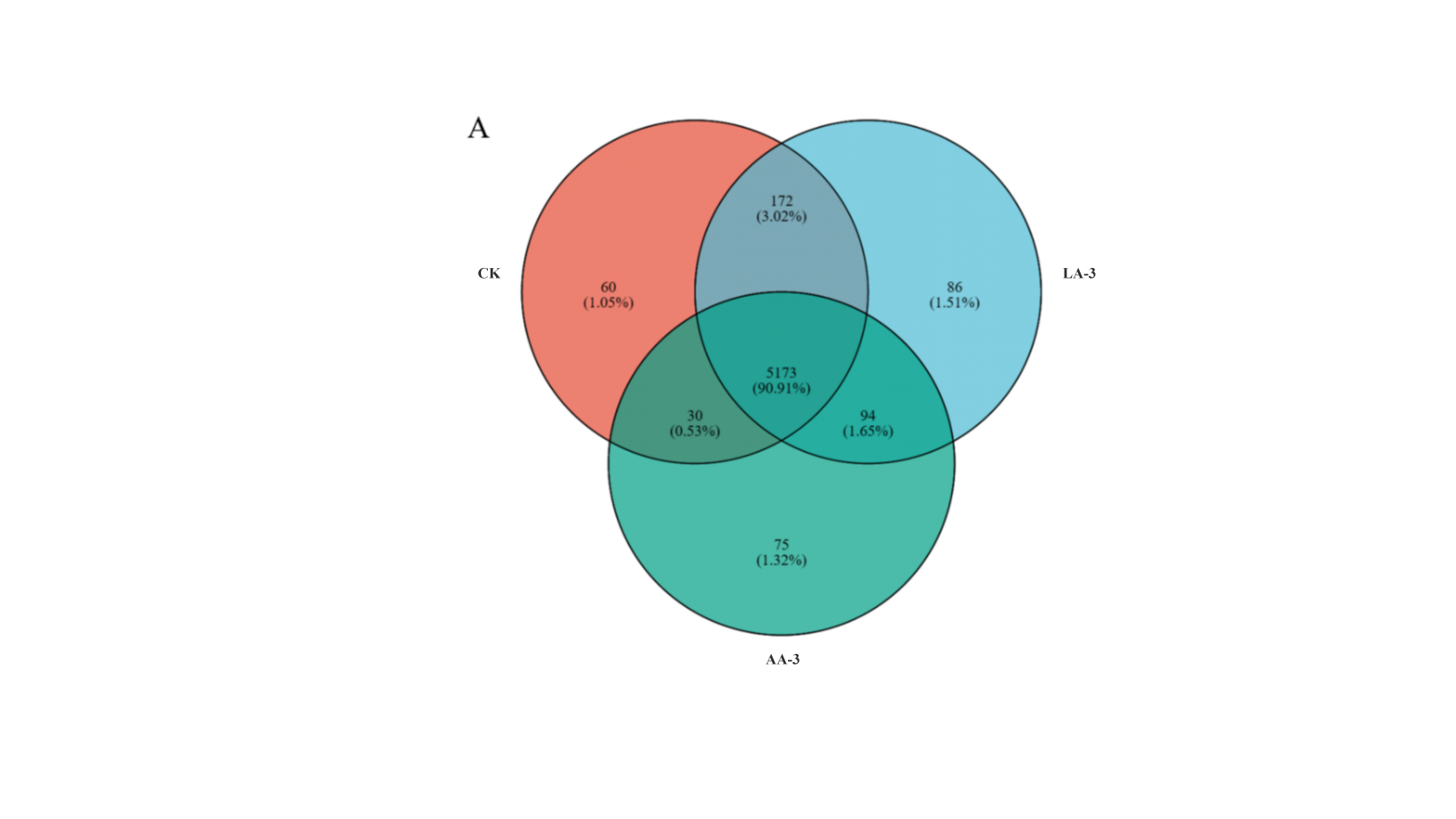
**

**Figure S3 Venn diagram (A) and volcano plots (B: acetic acid added; C: Lactate added)**


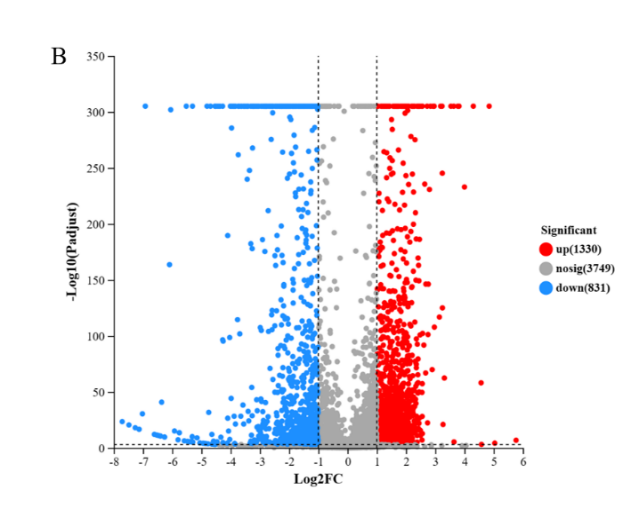

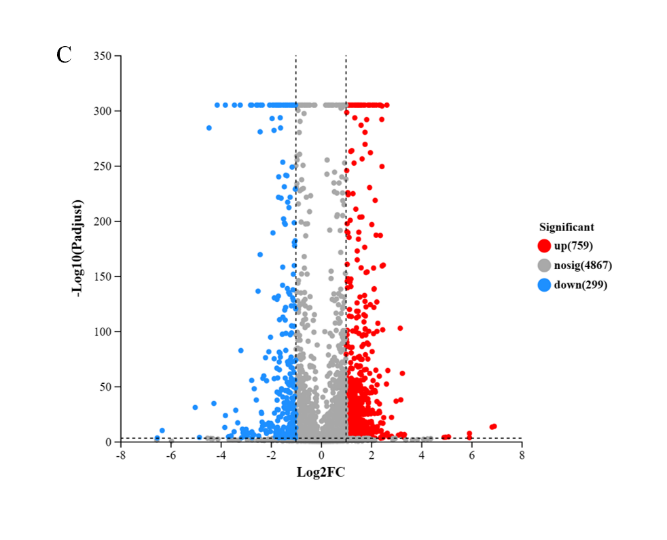


Note: In B, red indicates up-regulation, blue indicates down-regulation, gray indicates no significant difference

**Figure S4 KEGG functional annotation diagram of differential genes after fermentation with acetic acid (A) and lactic acid (B)**


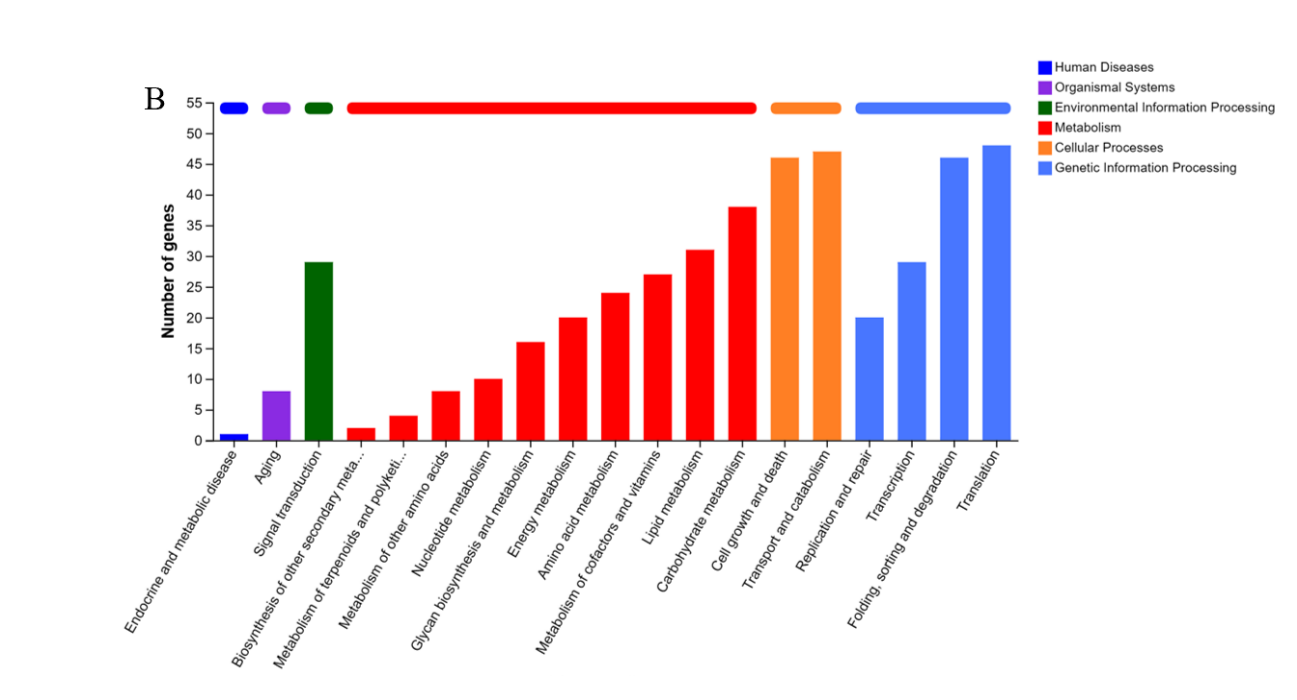

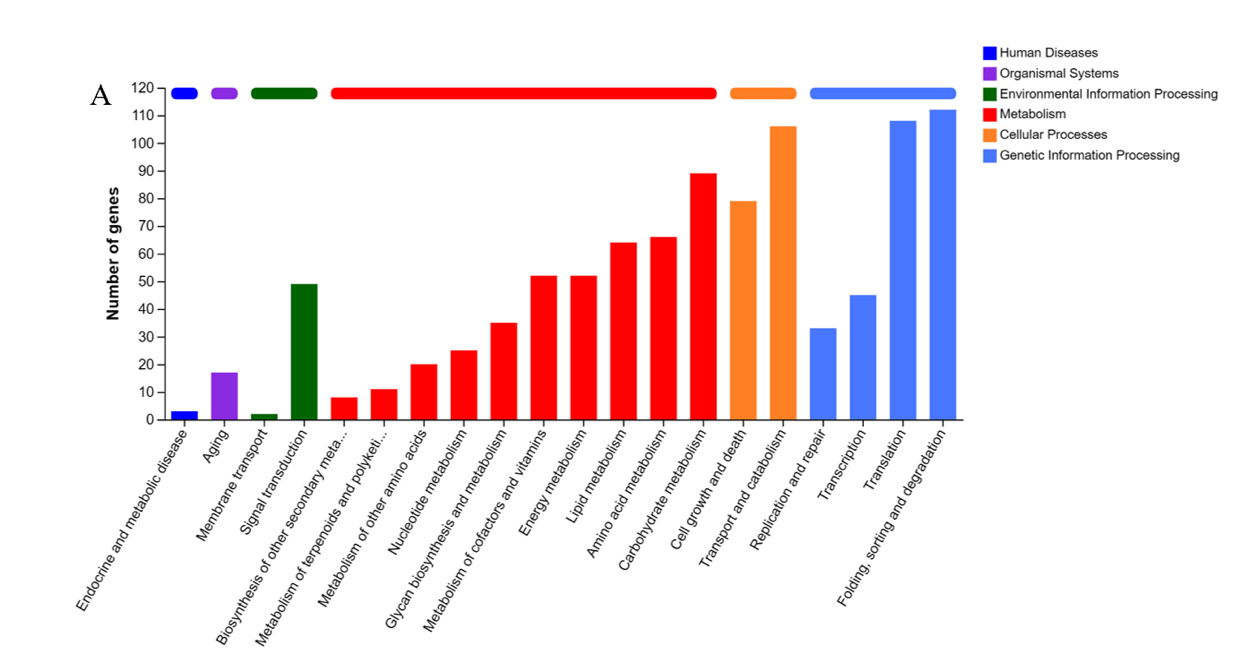

Supplement: Supplementary file 1 — Supplementary material [file mmc1.docx]
